# The use of the differential outcomes procedure for the recognition of facial expressions of complex emotions and its electrophysiological correlates

**DOI:** 10.3389/fpsyg.2024.1421688

**Published:** 2024-12-11

**Authors:** Ángel García-Pérez, Isabel Carmona, Angeles F. Estévez

**Affiliations:** ^1^Department of Psychology, University of Almería, Almería, Spain; ^2^Centro de investigación para el Bienestar y la Inclusión Social (CIBIS) Research Centre, University of Almería, Almería, Spain; ^3^Centro de Investigación en Salud (CEINSA) Health Research Centre, University of Almería, Almería, Spain

**Keywords:** electroencephalography, event-related potentials, differential outcomes, differential outcomes procedure, facial emotion recognition, complex emotions

## Abstract

The differential outcomes procedure (DOP) is an easily applicable method for enhancing discriminative learning and recognition memory. Its effectiveness in improving the recognition of facial expressions of emotion has been recently explored, with mixed success. This study aims to explore whether the expectancies generated via the DOP are reflected as differences in event-related potentials (ERPs) between participants in differential (DOP) or non-differential conditions (NOP) in a facial expression of complex emotion label task. Participants (*n* = 27 total, 14 DOP) in the DOP group received a specific reward for each specific emotion, while those in the NOP group received a random reinforcer when they correctly identified the emotion. We did not find differences in participants' accuracy or reaction time depending on group (DOP or NOP). These findings suggest that the DOP may not provide significant benefits for tasks involving labeling complex emotional expressions. However, differences in ERP components were observed between both groups. Specifically, the NOP group showed an increased Late Positive Component during encoding, fronto-central P300 during memory maintenance of facial stimuli, and frontal, fronto-central, and central P300 during retrieval. These ERPs, taken together, suggest that the task was more attentionally demanding for the NOP group. Additionally, some markers identified in previous ERP studies on the DOP were absent, indicating that the outcome expectancies may not have been fully generated. Finally, there were also interactions between the valence of the facial stimuli, participant group, and some of the potentials, such as N100 or N200 during encoding. These findings suggest that participants in the DOP group may have allocated more attentional resources to processing expressions of positive-valence emotions during earlier stages, possibly due to reward expectancy effects.

## 1 Introduction

Do expectations about the consequences of our actions influence processes such as learning or memory? In recent years, various studies have attempted to address this issue using the differential outcomes procedure (DOP) (Trapold, 1970). The DOP is a method used in conditional discrimination tasks, where specific reinforcers are paired with each correct stimulus-response association, typically in forced choice tasks, instead of administering any reinforcer per correct response, as in the non-differential outcomes procedure (NOP) (Fuentes et al., 2020). For instance, a specific reinforcer would be given if a child crosses the street when the traffic light is green, and a different one if they refrain from crossing the street when the light is red. This simple method was first introduced in non-human animal research (Goeters et al., 1992), where it improved discriminative learning (Mateos et al., 2016). Subsequently, studies have been conducted in humans, revealing faster learning and retention in discriminative training in children (Esteban et al., 2014; Estévez et al., 2001), young adults (Molina et al., 2015; Plaza et al., 2011), and older adults (Plaza et al., 2018) after applying the DOP. McCormack et al. (2019) highlight in their meta-analysis that these improvements exhibit effect sizes ranging from medium to medium-large.

The proposed mechanism underlying these enhancements involves the generation of unique outcome expectancies that are activated when the stimulus is detected via an implicit stimulus-outcome Pavlovian association (Carmona et al., 2019; Maki et al., 1995). According to the affective-associative two-process theory (for an overview, see Lowe et al., 2016; Lowe and Billing, 2017), non-differential outcomes generate typical operant stimulus-response pairings that require the maintenance in memory of the stimulus (retrospective route), while the use of differential outcomes allows for a stimulus-expectancy-outcome association that provides additional information (prospective route). Previous findings support that these implicit associations are generated regardless of stimuli awareness (Carmona et al., 2019) and that the two-routes, retrospective and prospective, engage different brain mechanisms (Savage, 2004, 2001). Mok et al. (2009) report fMRI results that support that these expectancies are generated when differential outcomes are awarded, as brain regions related to the modality of the outcomes (auditory or visual) are activated during delay periods that follow outcome presentation, when no stimuli are present. By contrast, when outcomes are non-differentially administered, only the hippocampus was activated. Recent electrophysiological studies (Carmona et al., 2020b,a) also show effects of the DOP in visuospatial memory encoding, maintenance, and retrieval, even without conscious processing of the outcomes. These effects were measured as differences in the N200 and P300 components during the encoding phase, the P300 component and positive-negative slow wave during the maintenance phase, and the N100 and P300 components during retrieval. Specifically, Carmona et al. (2020a,b) argue that P300 increases in the encoding phase can represent the activation of reward cues, and as such are heightened when differential outcomes are presented. They also report, during this same phase, that N200 may imply memory readouts of the stimulus-outcome pairings. P300 or Positive Slow Wave (PSW) increases during the maintenance phase can correspond to the activation of the representations of the outcomes while Negative Slow Wave (NSW), observed only in the non-differential group, involves the maintenance of the sample stimuli. Lastly, they also add that N100 effects during the retrieval phase suggest heightened top-down control in the non-differential group, and larger centroparietal P300 for participants in the DOP group hints of more outcome-related activation while the target stimuli are still being shown and during retrieval.

There have been recent attempts to study whether the DOP is also useful for the improvement of the recognition of facial expressions of emotion (González-Rodríguez et al., 2024, 2020) where, in the DOP condition, each of the reinforcers were associated with the emotion that the sample stimulus was to be labeled as. Both studies assessed young adults' performance in tasks employing pictures or videos of faces showing basic facial expressions of emotion and reported improvements in the recognition of a select few conditions (e.g., when participants must recognize expressions of fear and surprise). The authors discussed that the high accuracy of participants might indicate ceiling effects, with tasks not being challenging enough for them. As the effect of the DOP depends on the difficulty of the tasks with respect to participants (McCormack et al., 2019), and participants can present lower accuracies when labeling facial expressions of complex emotions (e.g., 75% mean correct responses for basic emotions vs. 62% mean accuracy for complex emotions in Benda and Scherf (2020)), assessing the recognition of non-basic emotions while using the DOP could mitigate this potential ceiling effect, thereby broadening the potential applications of this procedure. In a design similar to that of Carmona et al. (2020a,b), but employing a facial expression of emotion task, we would expect several of the ERPs that they report to appear here as well. Specifically, we anticipate observing we would expect to also find a P300 during encoding, as this component has been argued to reflect the orientation of attention toward task-relevant stimuli (for an overview, see Polich, 2007), and thus should appear when participants activate the representation of outcomes when they see the facial stimulus. We also expect to find late positive components (LPC), as both the P300 and the LPC have been linked to activation of motivational systems related to outcome processing (e.g., rewards) or emotional content of stimuli (Hajcak and Foti, 2020). Additionally, the N200 component reported by Carmona et al. should also be found during encoding, as it has been observed in studies involving the processing of facial expressions of emotion (e.g., Balconi and Pozzoli, 2012). Furthermore, during retrieval, we also expect that participants show a frontal N100 component, given its association with attention reorientation (Lange and Schnuerch, 2014), as the possible emotion label task will require participants to redirect attention to one of several stimuli.

Therefore, the purpose of this study is 2-fold. Firstly, we attempt to assess, for the first time to our knowledge, whether the DOP can be employed to improve the recognition of facial expressions of complex emotions in static pictures. Secondly, given the scarcity of studies investigating the electrophysiological correlates of the DOP, we also aim to further explore the time course of cognitive processes underlying this procedure. Specifically, we aim to utilize event-related potentials (ERPs) to study whether the unique differential associations between the outcome and the emotion are reflected in the same DOP-related ERP components reported in the two previous electrophysiological studies that employed an object recognition task (Carmona et al., 2020a,b). To explore this, we have added a delay period to a facial expression of complex emotion recognition task. We expect to find differences between participants subject to differential outcomes and participants receiving non-differential outcomes in the N200 and the late P300 (LPC) components during the encoding, the P300, PSW and NSW components during the maintenance, and the N100 and the P300 components during the retrieval phase (Carmona et al., 2020b).

## 2 Materials and methods

### 2.1 Participants

An a-priori power analysis was conducted using G^*^Power 3.1.9.7 (Faul et al., 2007) for a mixed ANOVA with Group (DOP or NOP) as between-subject variable and Emotion (Affectionate, Attracted, Betrayed, Brokenhearted, Contemptuous, and Desirous) as within-subject variable. The analysis employed α = 0.05, power = 0.80, and a medium-to-large effect size ($\eta_{\text{p}}^{2}$ = 0.1), which is about the typical effect size found in tasks that involve the DOP (McCormack et al., 2019). We set the correlation among repeated measurements to 0, as most accuracy correlations between emotions in an unpublished behavioral pilot study were non-significant. The resulting sample size calculation was *n* = 22. An additional power analysis was conducted for the ERP data employing a mixed ANOVA with Group (DOP and NOP) as a between-subject factor and Valence (Positive and Negative) and Hemisphere (Left and Right) as within-subject factors. With α = 0.05, power = 0.80, and assuming a medium-to-large effect size, the analysis showed a required sample size of *n* = 24.

All participants were Spanish-speaking, had normal or corrected-to-normal vision, and were young adults as there may be age effects on the recognition of facial expressions of emotion between observer and model (Hauschild et al., 2020), and the models in the employed database were also young adults (for more information on stimuli see below). Undergraduate students (DOP *n* = 14; 11 women, NOP *n* = 13; 10 women) between 18 and 25 years of age (DOP *mean* = 18.57, DOP *SD* = 0.64, NOP mean = 19.23, NOP SD = 2.04) were selected by convenience sampling and received one course credit for participating in the experiment. All participants gave their informed consent before participating in the experiment, which was conducted following the Declaration of Helsinki and received approval from the local bioethics committee.

### 2.2 Facial expression of complex emotion recognition label task

Black and white pictures of men and women from the Complex Emotion Expression Database (CEED; Benda and Scherf, 2020) of facial expressions of emotion that featured both open and closed mouth images were selected. Of the 9 complex emotions of the database Affectionate, Attracted, Betrayed, Brokenhearted, Contemptuous, and Desirous were selected. The task was designed using E-Prime 3.0 (Psychology Software Tools, Inc., 2016), which registered participant accuracy and reaction time. The screen background was set to gray, and sample stimuli were about 8° by 6° so that they resembled the size that faces occupy in the visual field at about 2 meters distance (Vehlen et al., 2022). The outcomes for DOP and NOP groups were pictures of a USB flash drive, a color-shifting speaker, earphones, a rucksack, a phone tripod/selfie stick, and a cell phone armband. The actual physical objects were raffled at the end of the study.

### 2.3 Procedure

Prospective participants were informed about the experiment via the local e-learning tool and were able to book appointments in an online calendar. After they arrived at their designated time and gave their written informed consent, they were sequentially assigned to either the NOP group or the DOP group. Then, they were led to an individual sound-attenuated experimental room. Before starting the experiment, they were instructed to minimize blinking and head movements while completing the facial expression of complex emotion recognition task. This task, which lasted for 35 to 40 min, consisted of a no-feedback 6-trial practice block followed by 2 120-trial blocks (20 trials per emotion in each block).

**Figure 1:**
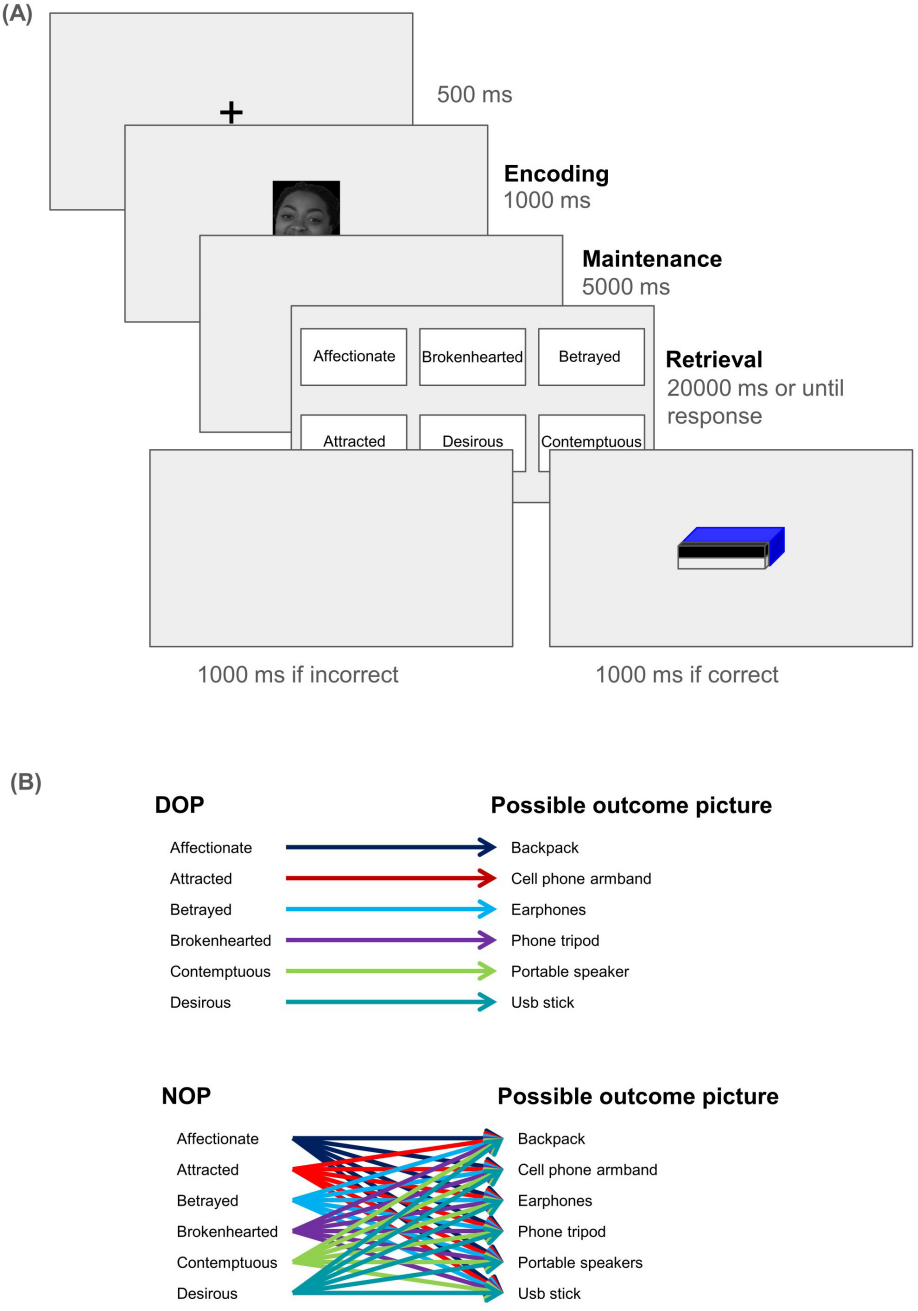
**(A)** Schematic representation of the stimulus sequence (from left to right). The three distinct phases for EEG analysis are bolded: participants saw the face (Encoding), which then disappeared, and the screen remained blank for 5 seconds (Maintenance). Lastly, participants had up to 20 seconds to select the label that best represented the emotion they saw (Retrieval). **(B)** Shows an example of the pairings between image and emotion label for the two experimental conditions (DOP and NOP). Those in the DOP condition always saw the same image for the same emotion, while those in the NOP condition would see any of the pictures when they correctly responded. To avoid systematic effects of reinforcer-emotion pairs, they were randomized across participants (e.g., one participant in the DOP may always see the picture of a backpack in correct Desire trials, while another may see the picture of a portable speaker).

At the beginning of the task, prior to the no-feedback practice trials, participants were presented with the following instructions in Spanish: “Thank you for participating in this experiment! In this experiment, pictures of faces showing a complex emotion (Affectionate, Attracted, Betrayed, Brokenhearted, Contemptuous, and Desirous) will appear. A few seconds later, the names of these emotions will appear. You must click with the mouse on the name of the emotion that you believe the face is expressing. After clicking on a name, the next trial will begin. There is only one correct response each time you see the names. You will not be informed whether your response was correct. Click to continue.”. These instructions were also verbally indicated to participants before they started the experiment. After the practice block, the same instructions appeared, but this time specifying that they would see the image of a prize that would be raffled if they responded correctly, and that the higher their accuracy, the higher their chances of winning the raffle. No image appeared on the screen if participants committed a mistake. Each trial began with a fixation point displayed for 500 ms, followed by the presentation of the facial stimulus for 1,000 ms (see Figure 1). Then, a blank screen was presented during 5,000 ms, after which the 6 complex emotion labels (Affectionate, Attracted, Betrayed, Brokenhearted, Contemptuous, and Desirous) appeared. After participants clicked one of the labels, or 20,000 ms had passed, either the prize (if they were correct) or a blank screen (if they failed to identify the emotion) appeared for 1,000 ms. Participants in the DOP condition always saw the same image for a specific emotion (emotion-prize pairings were randomized across participants), while those in the NOP condition saw a randomly selected prize when they correctly responded. For instance, a participant in the DOP group might always see the image of an USB flash drive if they correctly responded to Desire. The viewing distance was about 60 cm.

### 2.4 Electrophysiological data recording and processing

A 30-channel electrode cap (actiCAP, Brain Products GmbH, Munich, Germany) was used for electrophysiological recording, with standard 10–10 electrode location system, midfrontal electrode reference channel (FCz), grounding channel between FPz and FCz, and vertical and horizontal electrooculogram (VEOG and HEOG) placed 1 cm below the right orbital ridge and 1 cm lateral from the external canthi of the left eye. In addition, two electrodes were placed at left and right mastoid location to be able to re-reference the EEG data off-line to averaged mastoids. Electrode impedance was kept below 5 kΩ.

Electrode signal was amplified using a 32-channel AC-coupled amplifier (actiCAP, Brain Products GmbH, Munich, Germany) with a 250 Hz sampling rate (0.1–70 Hz band-pass, 50 Hz notch filter), digitally band-pass filtered (high cutoff: 25 Hz, 24 dB/octave attenuation; low cutoff: 0.1 Hz, 12 dB/octave attenuation), and was processed using the Brainvision Analyzer 2.0 software (Brain Products GmbH, Munich, Germany).

The EEG data were corrected for ocular/blink artifacts using Independent Component Analysis (ICA; Makeig et al., 1997). Then, data of correct responses were segmented from 100 ms before initial (face) stimulus onset to the response screen onset (6,100 ms). The EEG segments were corrected to a 100 ms baseline before the onset of the initial face stimulus. The EEG segments from the retrieval phase (from 100 before the onset of the response screen to 1,500 ms after it) were corrected to a 100 ms baseline before the onset of the response screen. Artifacts in each EEG segment and channel were automatically rejected (±100 μV maximal allowed amplitude; 50 μV maximal allowed voltage step; 200 μV maximal allowed difference of values in intervals; 0.5 μV lowest allowed activity; 100 ms interval length). EEG signals in all channels were re-referenced off-line to averaged mastoids before the segments were averaged. The number of averaged segments was >60 (>48% of valid trials) under all conditions.

To ensure a sufficient number of valid segments for EEG analysis, data were grouped according to the emotional valence, three emotions with positive valence (Affectionate, Attracted, and Desirous) and three with negative valence (Betrayed, Brokenhearted, Contemptuous), based on Benda and Scherf (2020).

Following the previous studies that measured ERPs while using the DOP (Carmona et al., 2020a,b), Frontal (F), Fronto central (FC), and Central (C) sites were analyzed. Centro-parietal and parietal sites (CP5, P3, P7, CP6, P4, and P8) were also initially examined, but no significant differences were found. This was partially expected since the task in this study was not visuospatial in nature, unlike those in previous studies. For analyses of all electrode sites and time windows, see https://osf.io/thxdj/?view_only=5001820878d84bafac624566fc859616. For ERP component detection, following the same studies, the trials were segmented into Encoding (from facial stimulus onset until disappearance; 0–1,000 ms), Maintenance (comprising the 5 seconds without any stimuli on the screen; 1,000–6,000 ms), and Retrieval (from label onset until 1,500 ms). In the encoding phase, the N100 component (50 ms to 150 ms), the N200 component (250 ms to 350 ms) and the Late Positive Complex (LPC or delayed P300; 600 ms to 850 ms) were analyzed in FC sites (FC1 and FC2). In the maintenance phase, the P300 component (1,250 ms to 1,400 ms) and the Positive and Negative Slow Waves (PSW-NSW, 3,100 ms to 5,900 ms) were assessed again in FC sites. Lastly, the N100 component (50 ms to 150 ms after label onset) and the P300 component (250 ms to 400 ms after label onset) were analyzed during the retrieval phase in three regions: F (F3, Fz, F4), FC (FC1, FCz, FC2), and C (C3, Cz, C4) regions.

## 3 Results

Demographic and raw behavioral data can be accessed at https://osf.io/thxdj/?view_only=5001820878d84bafac624566fc859616.

### 3.1 Behavioral data

#### 3.1.1 Accuracy

To analyze participants' accuracy, we conducted a mixed ANOVA with Group (DOP or NOP) as between-subject factor and Emotion (Affectionate, Attracted, Betrayed, Brokenhearted, Contemptuous, and Desirous) as between-subject factor. Table 1 depicts participants' accuracy in the task. There was no effect of Group, *F*_(1, 25)_ = 3.36, *p* = 0.078, nor a Group^*^Emotion interaction, *F*_(5, 25)_ = 0.93, *p* = 0.461, but there was an effect of Emotion, *F*_(5, 25)_ = 5.81, p < 0.0001, $\eta_{p}^{2}$ = 0.188. Specifically, participants responded better to Affectionate than Brokenhearted (*p* = 0.001) or Desirous (*p* = 0.001). No other pairwise comparison was found (all ps > 0.05).

**Table 1:** Means (and standard deviations) of participants' performance by Group and Emotion.

	**Aff**	**Att**	**Bet**	**Bro**	**Con**	**Des**
DOP	0.61 (0.25)	0.50 (0.22)	0.53 (0.13)	0.48 (0.13)	0.49 (0.25)	0.49 (0.16)
NOP	0.74 (0.13)	0.60 (0.16)	0.63 (0.15)	0.51 (0.15)	0.61 (0.16)	0.49 (0.13)

Aff, Affectionate; Att, Attracted; Bet, Betrayed; Bro, Brokenhearted; Con, Contemptuous; Des, Desirous; DOP, participants of the DOP group; NOP, participants of the NOP group.

Table 2 shows pairwise comparisons between emotions.

**Table 2:** Pairwise difference (column minus row) of means (and standard error) of participants' accuracy between emotions.

	**Aff**	**Att**	**Bet**	**Bro**	**Con**	**Des**
Aff						
Att	0.125 (0.043) *p =* 0.106					
Bet	0.094 (0.036) *p =* 0.216	−0.031 (0.036) *p =* 1	.			
Bro	**0.181 (0.037)** ***p** **=*** **0.001**	0.056 (0.042) *p =* 1	0.087 (0.030) *p =* 0.126			
Con	0.126 (0.045) *p =* 0.145	0.001 (0.045) *p =* 1	0.032 (0.044) *p =* 1	−0.055 (0.046) *p =* 1		
Des	**0.189 (0.039)** ***p** **=*** **0.001**	0.064 (0.041) *p =* 1	0.095 (0.033) *p =* 0.133	0.008 (0.040) *p =* 1	0.063 (0.044) *p =* 1	

Aff, Affectionate; Att, Attracted; Bet, Betrayed; Bro, Brokenhearted; Con, Contemptuous; Des, Desirous. Significant differences (*p* < 0.05) are bolded.

#### 3.1.2 Reaction time

Reaction time of correct responses was also analyzed. Reaction time data points further than 2 SDs from the mean were excluded (Berger and Kiefer, 2021). Table 3 depicts participants' reaction times in the task after filtering.

**Table 3:** Means (and standard deviations) of participants' reaction time by Group and Emotion.

	**Aff**	**Att**	**Bet**	**Bro**	**Con**	**Des**
DOP	978.83 (353.51)	941.37 (321.68)	1,027.10 (479.54)	933.44 (356.92)	1,135.20 (555.01)	841.79 (381.26)
NOP	693.29 (183.90)	772.34 (255.33)	729.75 (241.15)	812.62 (234.08)	891.58 (315.90)	712.05 (217.78)

Aff, Affectionate; Att, Attracted; Bet, Betrayed; Bro, Brokenhearted; Con, Contemptuous; Des, Desirous; DOP, participants of the DOP group; NOP, participants of the NOP group.

Similar to our accuracy analysis, we also conducted a mixed ANOVA with two between-subject factors Group (DOP or NOP) and Emotion (Affectionate, Attracted, Betrayed, Brokenhearted, Contemptuous, and Desirous) on reaction time means. There was no effect of Group, *F*_(1, 25)_ = 3.56, *p* = 0.071, and, as with accuracy data, no Group^*^Emotion interaction, *F*_(5, 25)_ = 0.92, *p* = 0.469, but there was a main effect of Emotion, *F*_(5, 25)_ = 5.81, *p* = 0.004, $\eta_{p}^{2}$ = 0.129. However, no pairwise emotion difference was found (all ps > 0.05). Table 4 shows pairwise comparisons between emotions.

**Table 4:** Pairwise difference (column minus row) of means (and standard error) of participants' reaction time between emotions.

	**Aff**	**Att**	**Bet**	**Bro**	**Con**	**Des**
Aff						
Att	−20.796 (42.551) *p =* 1					
Bet	−42.366 (53.730) *p =* 1	−21.570 (61.186) *p =* 1				
Bro	−36.973 (46.719) *p =* 1	−16.178 (47.634) *p =* 1	5.392 (46.108 *p =* 1			
Con	−177.333 (77.006) *p =* 0.448	−156.537 (71.512) *p =* 0.572	−134.967 (83.561) *p =* 1	−140.360 (63.455) *p =* 545		
Des	59.137 (45.441) *p =* 1	79.933 (38.242) *p =* 0.704	101.503 (43.440) *p =* 0.417	96.110 (42.483) *p =* 489	236.470 (73.139) *p =* 0.051	

Aff, Affectionate; Att, Attracted; Bet, Betrayed; Bro, Brokenhearted; Con, Contemptuous; Des, Desirous.

### 3.2 Electrophysiological data

For each of the ERPs stated above, a mixed ANOVA with Group (DOP and NOP) as between-subject factor, Valence (Positive and Negative) and Hemisphere (Left and Right) as within-subject factors for the Encoding and Maintenance phases and for N100 during Retrieval. For the P300 component in the Retrieval phase, an additional mixed ANOVA was performed with Region (Central, Frontal, and Fronto-Central), Laterality (Left, Middle, and Right), and Valence (Positive and Negative) as within-subject factors, and Group (DOP and NOP) as between-subject factor.

#### 3.2.1 Encoding, from 0 ms (facial stimulus onset) to 1,000 ms at fronto-central sites (left, FC1; and right, FC2)

**Figure 2:**
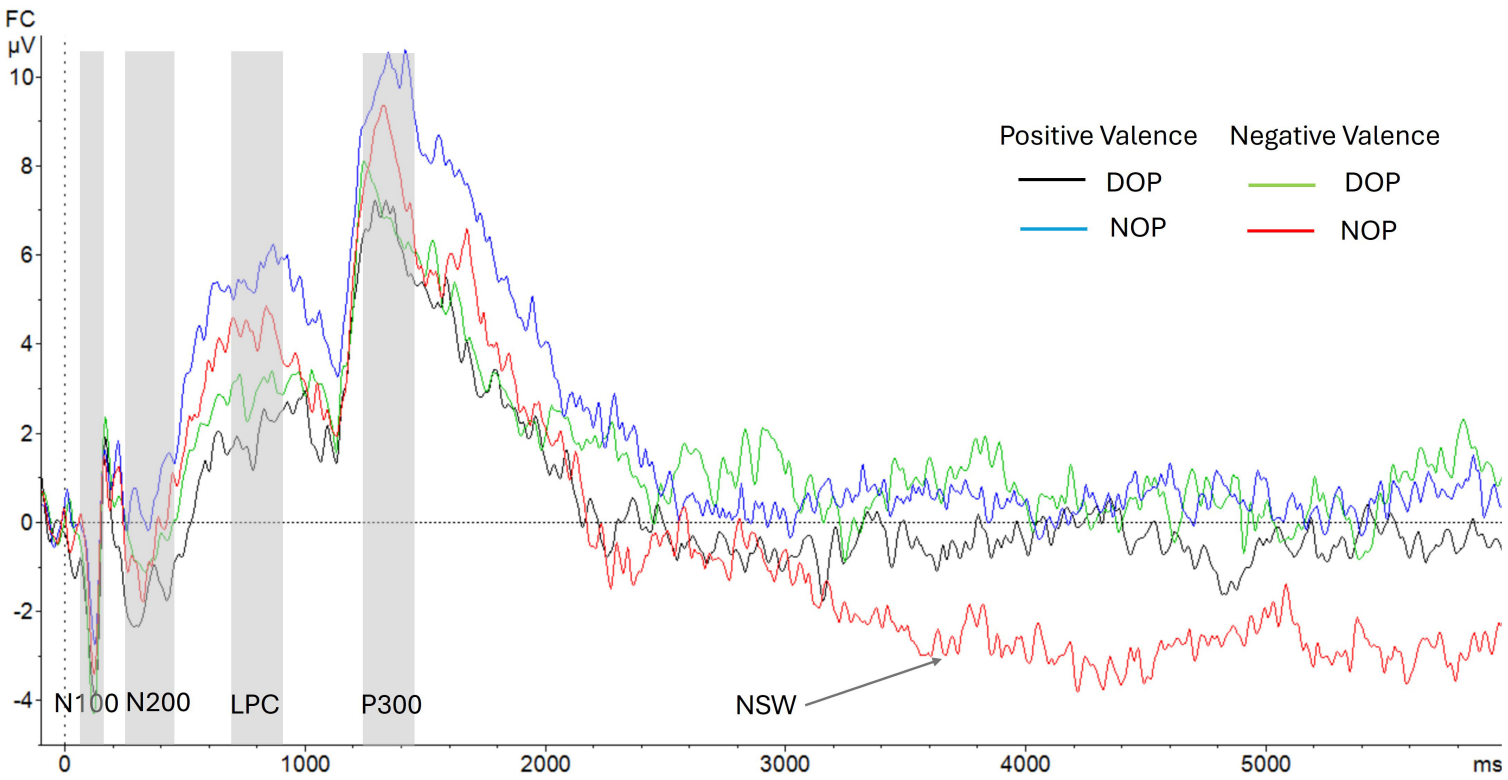
Grand-average voltage data (in μV) of ERP in the fronto-central region (FC: average of FC1 and FC2) as a function of Group and Valence (Positive Valence, differential –DOP, black line, vs. non-differential –NOP, blue line; Negative Valence, DOP, green line, vs. NOP, red line). Gray shades represent the N100, N200, LPC (Late Positive component), and P300 time windows. Time zero represents initial facial stimulus onset. The delay period between stimulus and labels includes the section from the vertical dotted line at 1,000 ms to 6,000 ms.

Figure 2 shows the ERP components analyzed in the encoding and the maintenance periods.

##### 3.2.1.1 N100 from 50 ms to 150 ms

**Figure 3:**
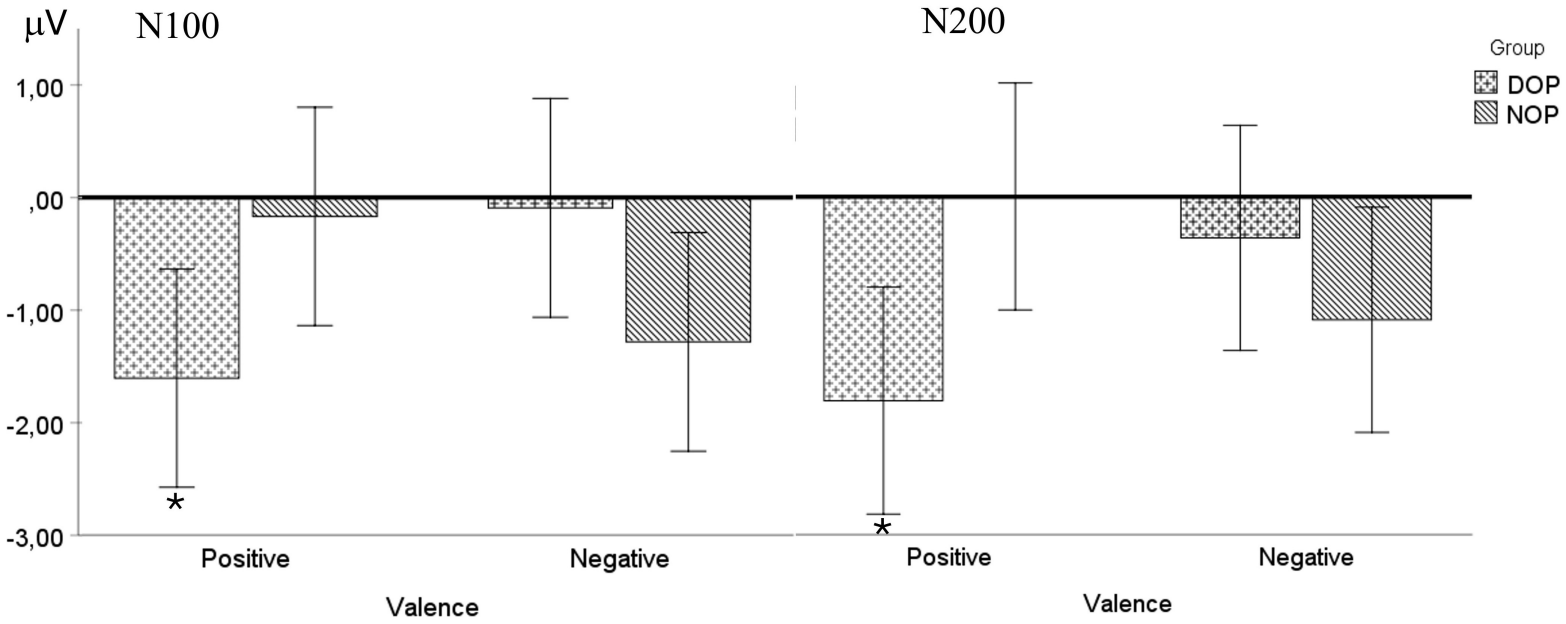
Mean voltage data (in μV) of the N100 **(right)** and N200 **(left)** components in the fronto-central region (FC: average of FC1 and FC2) as a function of Group (differential –DOP, and non-differential –NOP) and Valence (positive and negative). Asterisks indicate significant differences (*p* < 0.05).

Although the ANOVA (Valence X Hemisphere X Group) did not show any main effects [Fs < 1], a significant interaction between Valence and Group was found [*F*_(1, 24)_ = 11.4, *p* = 0.003, $\eta_{\text{p}}^{2}\;=$ 0.32]. Further analysis of this interaction indicated that this early ERP component was elicited only by emotions of positive valence in the DOP group [*t*_(12)_ = 3.15, *p* = 0.008; −1.73 mV, SD = 0.55]. By contrast, the N100 was absent to emotions of negative valence in both the DOP and the NOP groups (see Figure 3).

##### 3.2.1.2 N200 from 250 ms to 350 ms

Once again, the analysis did not show any main effects [Fs < 1].

Additionally, the analysis of this component also revealed a significant two-way Valence X Group interaction [*F*_(1, 24)_ = 8.8, *p* = 0.007, $\eta_{\text{p}}^{2}$ = 0.27]. Specifically, in the Positive Valence condition, the DOP group showed a larger N200 component (−1.80 μV) compared to the NOP group (0 μV), while in the Negative Valence condition, the NOP group showed greater voltage (−1.10 μV) compared to the DOP group (−0.37 mV).

Further analysis comparing the N200 amplitude with the baseline (0 μV) in each group showed that the voltage to emotions of negative valence was similar to the baseline amplitude in the DOP group [*t*_(12)_ = 0.51, *p* = 0.622] (for positive valence emotions, *t*_(12)_ = 3.17, *p* = .008) and both emotional valences in the NOP group [positive, *t*_(12)_ = 0.002, *p* = 0.99; Negative, *t*_(12)_ = 0.90, *p* = 0.38] were similar to the baseline amplitude (see Figure 3). No other interactions were observed (*p*s > 0.05).

##### 3.2.1.3 LPC (Late Positive Component or delayed P300) from 600 ms to 850 ms

The ANOVA showed a main effect of Group [*F*_(1, 24)_ = 7.32, *p* = 0.012, $\eta_{\text{p}}^{2}$ = 0.23]. The amplitude of the LPC was higher in the NOP group (5.5 μV; SD, 0.87) than in the DOP group (2.2 μV; SD, 0.87).

In addition, a two way interaction between Valence and Hemisphere was found [*F*_(1, 24)_ = 5.11, *p* = 0.033, $\eta_{p}^{2}$ = 0.18]. Further analysis of this interaction indicated no valence-based differences (*p*s > 0.05) between hemispheres (right vs. left hemisphere to positive valence, 3.3 μV vs. 3.8 μV; and to negative valence, 4.4 μV vs. 3.5 μV). No other main effects nor interactions were found (*p*s > 0.05).

#### 3.2.2 Maintenance, delay period from 1,000 ms after facial stimulus onset until 6,000 ms at fronto-central sites (left, FC1 and right, FC2)

##### 3.2.2.1 P300 from 1,250 ms to 1,400 ms

The ANOVA showed a main effect of Group [*F*_(1, 24)_ = 6.54, *p* = 0.017, $\eta_{\text{p}}^{2}$ = 0.21]. The P300 amplitude was higher in the NOP (5.8 μV; SD, 0.89) than in the DOP condition (2.9 μV; SD, 0.89). No other main effects nor interactions were found (*p*s > 0.05).

##### 3.2.2.2 NSW (Negative Slow Wave) from 3,100 ms to 5,900 ms

**Figure 4:**
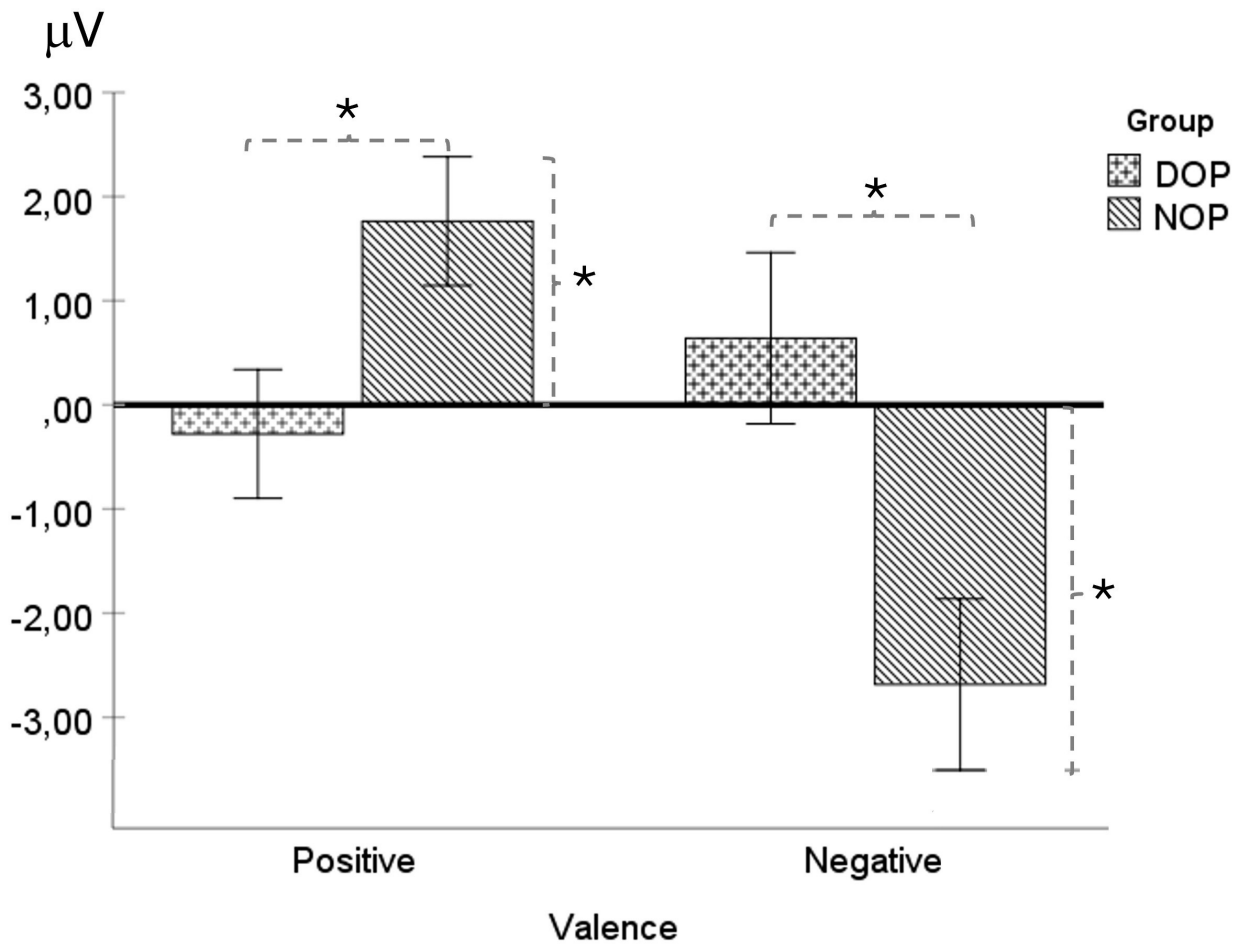
Mean voltage data (in μV) of the NSW (Negative Slow Wave) in the fronto-central region (FC: average of FC1 and FC2) as a function of Group (differential –DOP, and non-differential –NOP) and Valence (positive and negative). Simple asterisk means significant differences (*p* < 0.05).

There was a main effect of Valence [*F*_(1, 24)_ = 8.39 *p* = 0.008, $\eta_{p}^{2}$ = 0.26]. Specifically, the NSW amplitude was higher for emotions of negative (−1.02 μV, SD = 0.58) than for positive valence (0.75 μV, SD = 0.44). Moreover, the two-way interaction Valence X Group was statistically significant [*F*_(1, 24)_ = 19.4 *p* < 0.001, $\eta_{p}^{2}$ = 0.45]. Significant differences between groups were found for both positive valence emotions [*F*_(1, 24)_ = 5.46, *p* = 0.028; DOP, −0.28 μV, SD = 0.56, and NOP, 1.76 μV, SD = 0.67], and negative valence emotions [*F*_(1, 24)_ = 8.17, *p* = 0.009; DOP, 0.64 μV, SD = 0.76, and NOP, −2.68 μV, SD = 0.87]. Further analysis indicated that this wave was similar to the baseline (zero value) in the DOP for both valences, while the NSW was elicited in the NOP group for emotions of negative valence [*t*_(12)_ = 3.07, *p* = 0.01] (see Figure 4) and a Positive Slow Wave was registered for emotions of positive valence [*t*_(12)_ = 2.6, *p* = 0.023]. No other main effects or interactions were found [*F*s < 1].

#### 3.2.3 Retrieval: from 0 ms (response screen onset) to 1,500 ms at frontal (F3, Fz, F4), fronto central (FC1, FCz, FC2), and central (C3, Cz, C4) sites

##### 3.2.3.1 N100 from 50 ms to 150 ms and N200 from 150 ms to 250 ms

No effects were found in the ANOVA (Region X Laterality X Valence X Hemisphere X Group) for either component (all *F*s < 1).

##### 3.2.3.2 P300 from 250 ms to 400 ms

**Figure 5:**
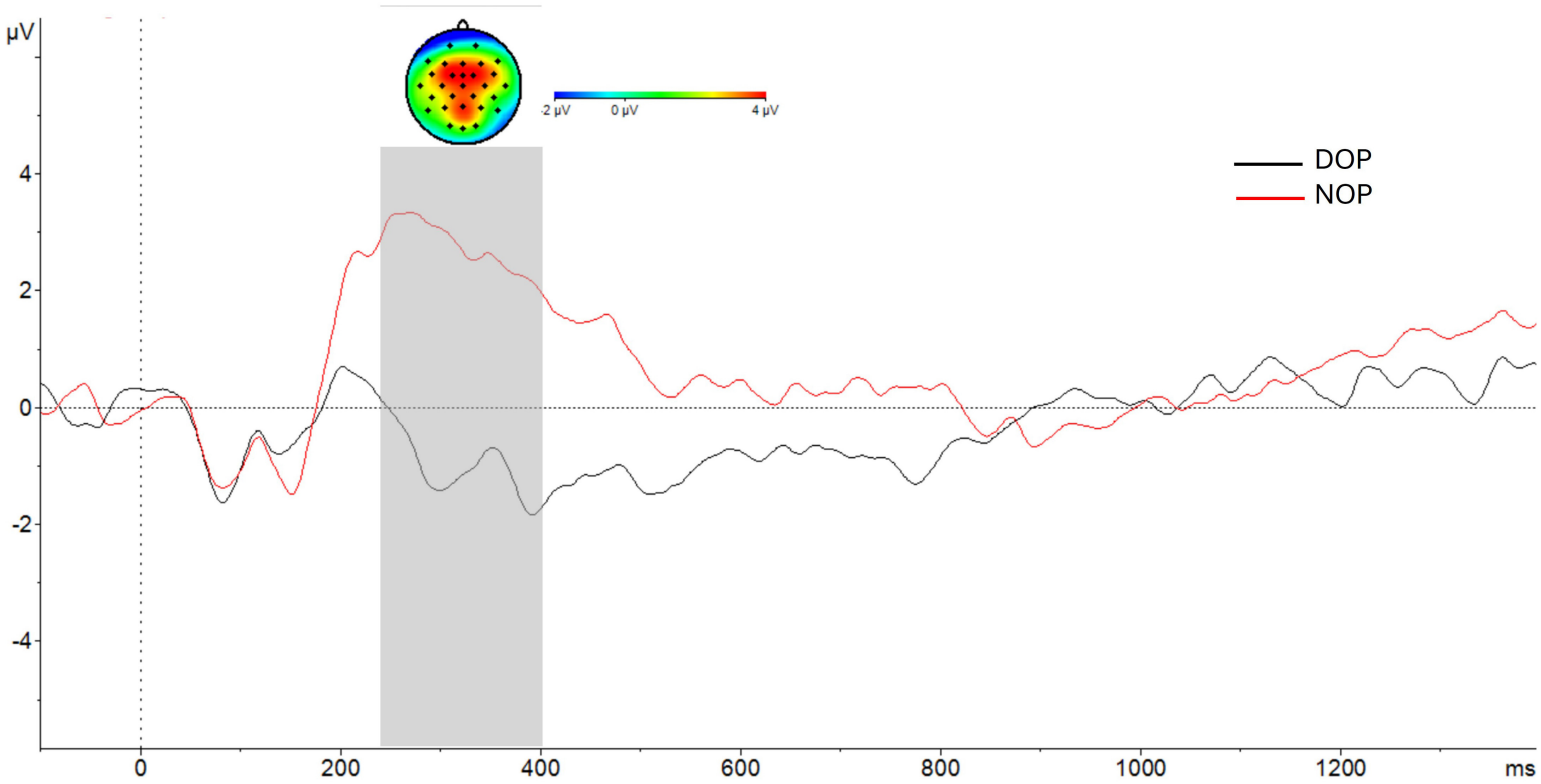
At the bottom, grand-average voltage data (in μV) of ERP in the frontal, fronto-central and central regions (average of F3, Fz, F4, FC1, FCz, FC2, C3,Cz, C4) as a function of Group (differential –DOP, black line, vs. non-differential –NOP, red line). Gray shades represent the P300 time window. At the top, topographic map of the difference in ERP waves between Groups (NOP–DOP) in the P300 time window. Time zero represents response screen-onset.

The ANOVA (Region X Laterality X Valence X Group) showed a main effect of Group [*F*_(1, 24)_ = 11.5, *p* = 0.002, $\eta_{p}^{2}$ = 0.32]. The amplitude of the P300 component was higher in the NOP (2.8 μV; SD, 0.89) than in the DOP condition (-0.77 μV; SD, 0.89) as can be seen in Figure 5.

In addition, the Region factor [*F*_(1, 24)_ = 14.14, *p* < 0.001, $\eta_{p}^{2}$ = 0.37] and the Laterality factor [*F*_(1, 24)_ = 5.7, *p* = 0.006, $\eta_{p}^{2}$ = 0.19] reached statistical significance. Table 5 summarizes the voltage data as a function of Region and Laterality. Regarding the Region factor, significant differences in P300 amplitude were found between the Central site and the other two regions, Frontal (*p* = 0.002) and Fronto-central (*p* = 0.001).

**Table 5:** Mean voltage data (in mV) as a function of Region (Frontal, Fronto central, and Central) and Laterality (left, middle, and right).

**Region**	**Mean (mV)**	**SD**	**Laterality**	**Mean (mV)**	**SD**
Frontal	0.25	0.58	Left	0.63	0.63
Fronto-central	0.83	0.59	Middle	0.73	0.56
Central	2.01	0.52	Right	1.73	0.52

SD, standard deviation.

Finally, the P300 amplitude (see Table 5) in the right hemisphere was significantly higher than in the left hemisphere (*p* = 0.037) and in the middle axis (*p* = 0.014). No other main effects nor interactions were found (*p*s > 0.05).

## 4 Discussion

This study was conducted to explore both the potential utility of the DOP in enhancing delayed recognition of complex emotions in static faces and the time course of cognitive processes underlying this procedure by using electroencephalography to measure previously reported DOP-related ERPs.

Regarding our first objective, we found no behavioral effect of the DOP on either reaction times or accuracy, although we did find an effect of emotion, with Affectionate being easier to recognize than two other emotions (Brokenhearted and Desirous). These findings are somewhat expected, as, despite McCormack et al. (2019) finding a medium to large effect size of studies employing the DOP in their meta-analysis, there are mixed results concerning the DOP in the few studies that have been published on the recognition of facial expressions of emotion (González-Rodríguez et al., 2024, 2020). This lack of effect of the DOP on participant performance could result from the pairing of the outcomes with expressions of emotion. Specifically, the DOP generates expectancies based on the association between the outcomes and the sample stimuli (Trapold, 1970), and the configuration of facial expressions of emotion can vary for a given emotion (Barrett et al., 2019). As we have applied the same reinforcer for all pictures originally labeled as the same complex emotion in Benda and Scherf (2020) (e.g., the same reinforcer for all “affectionate” images) and the models may present slightly different emotional expressions, perhaps the expectancies are being generated for only a subset of the stimuli. Consequently, the reinforcement would not really be differential for all varying expressions of an emotion that a person might understand as such. To address this limitation, a different experimental procedure could be employed, swaying away from the classical label or match tasks that are frequently reported in the literature regarding recognition of facial expressions of emotion (Palermo et al., 2013), and instead pairing the representation of any facial expressions of a particular emotion that a participant has with an outcome. This could be achieved, for instance, by first presenting the name of an emotion, and then asking participants to select the picture containing an expression corresponding to that specific emotion instead of presenting the picture of the facial expression and then asking participants to correctly label them (a classic label task). Reinforcers in the DOP condition in this alternative task would also be associated with each of the labels. This way, we would expect participants to activate several facial emotion configuration representations that they may have stored for a given emotion. Another possibility is that the effect of the DOP for the recognition of facial expressions of emotion is smaller than we expected (e.g., it is not as effective for the recognition of facial expressions of emotion), which would mean that another study should be carried out employing a bigger sample size to replicate these findings while ensuring proper power for smaller effects.

Concerning our second objective, the event related potentials only partially matched those reported by Carmona et al. (2020b,a) during the encoding phase. Specifically, we also found differences between our DOP and NOP groups in the N200 frontocentral component as well as, for the first time, in the N100 component during encoding, but these differences interacted with stimuli valence. Balconi and Pozzoli (2012) found differences in the N200 ERP during the encoding phase in tasks involving either incidental or direct processing of facial expressions, which was modulated by the stimuli's arousal and valence values. Therefore, these differences based on stimuli valence are not entirely unexpected. They found that the N200 potential was stronger for negative emotions. This may be explained by the fact that negative emotion represents a situation that threatens our safety and, therefore, requires more attention (Ellsworth and Scherer, 2003). In contrast, positive emotions, such as happiness, indicate a low-threat situation, and therefore do not require increase of arousal and attention. The same pattern reported by Balconi and Pozzoli (2012) (greater deflection for negative than for positive valence) was observed in the NOP group, although the recorded activation in this case was similar to that of the baseline. Importantly, this trend was reversed in the DOP group, whose results resemble those found by Carmona et al. (2020b,a), showing a stronger N200 for positive emotions that differed from the baseline. This may suggest that the DOP group allocated more attentional resources during early stages of encoding for positive emotions. How could the influence of the way we present the outcomes in this early stage of attentional processing, particularly concerning positive emotions, be explained? To address this, we need to consider two factors. First, the N200 component is closely related to effects of reward expectancy effects (Gheza et al., 2018). Second, the task overall proved challenging for participants, as evidenced by their performance. Under such conditions, it has been found that more training sessions are needed for the beneficial effect of using the DOP to emerge (Molina et al., 2020). It is possible that, in the present study, although we employed a larger total number of trials than most studies included in McCormack et al. (2019)'s meta-analysis, the stimulus-outcomes association is starting to be established and generating the expectancies, which will need further training to consolidate. This could explain why no effects were observed at the behavioral level, yet some effects began to emerge at the electrophysiological level during the early stages of information processing, wherein the sample stimulus is evaluated and categorized—processes that can be influenced by reward expectancies. In our study, we may detect this component precisely with positive emotions, which seem to be somewhat easier to identify when considered together (57% vs. 54% accuracy and 823.28 vs. 921.62 response speed for positive vs. negative valence emotions, respectively). If this is the case, an increased number of training sessions could lead to a greater activation of the N200 component for both positive and negative valence emotions when employing the DOP, an effect that, moreover, could be evidenced in other components related to the activation of outcome expectancy across the different phases explored (encoding, maintenance, and retrieval). Future research is needed to address this question. To increase the number of training trials, and given the apparent differential processing of stimuli with varying emotional valence, these studies may also consider training each type of emotion separately. These studies should also consider the type of outcome used. In our case, we employed positive reinforcement, with rewards that are, in principle, associated with things participants may desire and are non-threatening. It might be that the generation and activation of the outcomes expectations are facilitated if the valence of the feedback stimuli matches that of the sample (facial) stimulus. However, further research is required to support this claim (e.g., designing an experiment using differential negative reinforcement and facial stimuli with positively or negative expressions, or a task employing neutral outcomes such as meaningless geometrical shapes).

It is also worth noting that, in the encoding phase, there was an additional difference compared to the potentials reported in the studies by Carmona et al. (2020b,a): differences in the LPC were also found, with NOP participants displaying a larger LPC. This component is related to emotional processing, with increases in LPC indicating deeper emotional processing and attentional resource allocation to emotional stimuli (Ding et al., 2017; Hajcak et al., 2009, 2010). Specifically, the LPC has been linked, during encoding, to the allocation of attentional resources toward task-relevant emotional stimuli during sustained attention (Schupp et al., 2006), at later stages of processing. This suggests that participants receiving non-differential outcomes engaged in deeper processing of stimuli, including sustained attention at later stages of processing, to achieve comparable performance in the task. This need for greater use of attentional resources during the task continues to be evident in the following phases, maintenance and retrieval.

During the maintenance phase, there were differences that again partially matched the findings in the two aforementioned studies. Specifically, we found higher frontocentral P300 amplitudes in our NOP group, and differences in the NSW component for all valences between the two groups. As in Carmona et al. (2020b)'s, the NOP group showed a NSW but only for negative emotions. In fact, a PSW for positive emotions was observed in this group. Furthermore, the DOP group did not differ from their baseline measurement. As Carmona et al. (2020a,b) argue that the maintenance NSW may reflect a stronger effort to keep the representations of the sample stimuli, while the PSW could be related to outcome processing, our results may imply that (i) the NOP group could be differently processing the positive and negative stimuli in order to solve the task and (ii) the DOP could not be properly generating the emotion-outcome expectancies or, as previously stated, more training is still required to consolidate the pairings. This again suggests that further studies may consider reversing the order of the stimuli and the labels in a new task, to ensure the outcomes are being paired with participants' wider repertory of representations of possible configurations for an emotion and/or, as previously mentioned, to increase the training. Interestingly, the fronto-central P300, which was only found in the NOP group, is related to dopaminergic attentional resources (Polich, 2007). This suggests that participants in the NOP group were also allocating a greater amount of attentional resources during this phase.

Our electrophysiological results for the retrieval phase also clash with those reported regarding the DOP. Specifically, we did not observe an N100 component, and also found a higher P300 amplitude in the NOP group, while the reverse was reported in all available DOP-ERP studies (Carmona et al., 2020a,b). As the P300 component is related to attentional mechanisms (Polich, 2007; Van Dinteren et al., 2014), this may reflect that the NOP group further engaged their attentional resources, which also aligns with their abovementioned higher P300 during the maintenance phase and with the stronger LPC (or delayed P300) during the encoding phase. Which may reflect deeper processing of the stimuli (Rellecke et al., 2012) during such a phase. It is important to note that we did not find any significant centro-parietal potentials, including centro-parietal P300 components, during retrieval or encoding. A heightened centro-parietal P300 in the DOP group during these two phases was one of the main findings of the two ERP studies mentioned, purportedly reflecting effects of reward expectancies. As we did not find any of these components, our results again suggest that the expectancies generated by the DOP may not have functioned as expected. However, throughout all three phases (encoding, maintenance, and retrieval), as stated above, we did find ERPs indicating that the NOP group may have needed to further employ attentional resources than the DOP group to complete the task.

The limitations of this study should be noted. Firstly, there is a reduced sample size, and the sample is composed entirely of healthy undergraduates that were not randomly selected, which might introduce selection biases, and also reduces the generalizability of our findings. Furthermore, there are a number of factors that reduce the ecological validity of our findings: (a) the introduction of a delay between the face pictures and the appearance of the emotion labels so that the task was more similar to most of the paradigms that involve the DOP, which usually feature a delay (McCormack et al., 2019), and also to detect the ERP correlates of the expectancies introduce memory effects, while the recognition of emotional states in others is usually performed while the social stimuli in question is present; and (b) the to-be-labeled stimuli were static pictures, while the recognition of emotional states in dynamic stimuli (e.g., such as in face-to-face interactions) is distinct to that of static pictures (Krumhuber et al., 2013). Additionally, out of the many possible facial expressions of complex emotional states that could be shown, only a subset of expressions of social sexual emotions were employed (Benda and Scherf, 2020). It is unknown whether any possible improvements generated by reinforcement do generalize to other emotional states that have not been actively trained (e.g., whether the recognition of emotional expressions itself is being enhanced or only the trained subset of facial stimuli or selected emotions), or whether similar findings would also be observed employing different emotional expressions, such as self-conscious emotions (Joanne and Richard, 2015).

In conclusion, the behavioral results of this study suggest that the DOP may not be particularly useful for improving the recognition of complex facial expressions of emotion in a label task. Contrastingly, the electrophysiological results indicate that participants might employ different processing pathways depending on how the outcomes are administered after their correct responses, differentially or non-differentially, even in the absence of behavioral effects. Therefore, further research is needed before providing a clear answer to this issue.

## Data Availability

The datasets presented in this study can be found in online repositories. The names of the repository/repositories and accession number(s) can be found below: https://osf.io/thxdj/?view_only=5001820878d84bafac624566fc859616.
